# Sequence Effect in Parkinson’s Disease Is Related to Motor Energetic Cost

**DOI:** 10.3389/fneur.2016.00083

**Published:** 2016-05-24

**Authors:** Sule Tinaz, Ajay S. Pillai, Mark Hallett

**Affiliations:** ^1^Department of Neurology, Division of Movement Disorders, Yale School of Medicine, New Haven, CT, USA; ^2^Human Motor Control Section, National Institute of Neurological Disorders and Stroke, National Institutes of Health, Bethesda, MD, USA

**Keywords:** bradykinesia, decrement, dopamine, visual feedback, cost–benefit, motor control, fatigue

## Abstract

Bradykinesia is the most disabling motor symptom of Parkinson’s disease (PD). The sequence effect (SE), a feature of bradykinesia, refers to the rapid decrement in amplitude and speed of repetitive movements (e.g., gait, handwriting) and is a major cause of morbidity in PD. Previous research has revealed mixed results regarding the role of dopaminergic treatment in the SE. However, external cueing has been shown to improve it. In this study, we aimed to characterize the SE systematically and relate this phenomenon to the energetic cost of movement within the context of cost–benefit framework of motor control. We used a dynamic isometric motor task with auditory pacing to assess the SE in motor output during a 15-s task segment in PD patients and matched controls. All participants performed the task with both hands, and without and with visual feedback (VF). Patients were also tested in “on”- and “off”-dopaminergic states. Patients in the “off” state did not show higher SE compared to controls, partly due to large variance in their performance. However, patients in the “on” state and in the absence of VF showed significantly higher SE compared to controls. Patients expended higher total motor energy compared to controls in all conditions and regardless of their medication status. In this experimental situation, the SE in PD is associated with the cumulative energetic cost of movement. Dopaminergic treatment, critical for internal triggering of movement, fails to maintain the motor vigor across responses. The high motor cost may be related to failure to incorporate limbic/motivational cues into the motor plan. VF may facilitate performance by shifting the driving of movement from internal to external or, alternatively, by functioning as a motivational cue.

## Introduction

Bradykinesia, one of the cardinal manifestations of Parkinson’s disease (PD), means slowness of movement. It is the most disabling symptom and a complex phenomenon with different components (1). In the clinical setting, bradykinesia is assessed during repetitive sequential movements (e.g., finger tapping). The amplitude and/or speed of the movements rapidly diminishes with each repetition. This phenomenon is known as the “sequence effect” (SE) and seems unique to PD among other parkinsonian syndromes (2, 3). The SE is an important source of morbidity in daily activities of PD patients, including handwriting, gait, and speech (3–5), for which dopaminergic treatment falls short. Several behavioral paradigms have been used to characterize the SE and reports on the role of dopaminergic treatment are heterogeneous. Some studies demonstrated that while certain components of bradykinesia responded well to dopaminergic treatment, the SE did not (6–8).

Although SE is a well-known clinical observation, the underlying pathophysiology has not been fully characterized. While one cannot exclude the contribution of peripheral fatigue to the SE, there are two observations that point to a central mechanism: (1) the onset of the decrement is rather abrupt occurring within seconds and (2) it can be reversed with visual or motivational cueing. Various central mechanisms for bradykinesia have been proposed that are also relevant in understanding the SE:

(1) Insufficient motor energy was considered an important factor in bradykinesia. Electrophysiological studies have demonstrated that movements are not given the full motor command that they require (9, 10) due to inadequate cortical drive to the muscle (11, 12). This deficit improves with dopaminergic treatment.
(2) Deficit in scaling was also thought to contribute to the underscaling of the desired movement (13–15).
(3) Bradykinesia and SE can also be considered part of “central fatigue” (16). Patients with central fatigue have difficulty with sustained performance in serial tasks. This is also a fundamental problem in PD and patients often report feeling as if their “battery is running down” (17).
(4) Finally, in recent years, behavioral studies and computational models have operationalized bradykinesia and related problems within the “cost–benefit” framework. According to this framework, PD patients assign implicitly (i.e., out of awareness) a higher energetic cost to a motor task, therefore, “scale down” their motor vigor (speed, amplitude, or force) as an implicit adaptive response in order to optimize motor effort (18–20). This suggests that PD patients are capable of demonstrating a motor performance comparable to that of controls, but at a higher cost, i.e., they would have to exert higher effort than controls.

In this study, we approached the SE as a central problem of motor energy and aimed to characterize it systematically using a repetitive motor task in two conditions: (1) patients and matched healthy volunteers (HVs) were tested using a demanding dynamic isometric task with and without visual feedback (VF) on their performance and (2) patients were also tested in “off”- and “on”-dopaminergic states.

We hypothesized that (1) PD patients in off-dopaminergic state will show significantly higher SE compared to HVs, (2) dopaminergic treatment will not improve SE, but VF will, and (3) the energetic cost of motor performance will be higher in patients compared to HVs regardless of medication status.

## Materials and Methods

### Participants

Thirteen right-handed PD patients and 13 right-handed age- and gender-matched HVs participated in the study after giving written informed consent in accordance with the Combined NeuroScience Institutional Review Board of the National Institutes of Health. Patients were recruited through the Parkinson’s Disease Clinic at the National Institute of Neurological Disorders and Stroke. One patient tested positive for the LRRK2 gene and was excluded. One HV was also excluded due to an error in data recording. The data of 12 PD subjects (5 F, average age 63.0 ± 6.4) and 12 HVs (6 F, average age 62.7 ± 6.9) were included in the analysis.

All participants underwent physical and neurological examinations. The following exclusion criteria applied to all participants: the presence of any neurological or psychiatric disorder (other than PD and comorbid depression or anxiety for the PD group), or a medical condition that might affect the central nervous system, and active alcohol or illicit drug abuse. The diagnosis of PD was established according to the UK Parkinson’s Disease Society Brain Bank Clinical Diagnosis Criteria (21). All patients had bradykinesia and at least one of the following impairments: rigidity, resting tremor, or postural instability. The side of disease onset was left in half, and right in the other half of patients. Patients were assessed using the Unified Parkinson’s Disease Rating Scale (UPDRS) (22) and the Hoehn and Yahr (H&Y) scale (23). Patients with a UPDRS tremor score >1 in either hand and with an H&Y score >3 were not included. Patients were tested first off of any dopaminergic medication in the morning. Immediately upon completion of the “off” testing, they were given their regular dose of levodopa/carbidopa and other dopaminergic medications, and tested again at the peak of their “on” state. The “off” state was defined as at least a 12-h washout period for immediate-release levodopa/carbidopa and dopamine receptor agonists, and 24 h washout for MAO-B inhibitors and extended-release formulations of levodopa/carbidopa and dopamine receptor agonists. All patients were responsive to levodopa/carbidopa (within 80 ± 34 min). The “on” state was established by patients’ subjective report and objective neurological exam. The levodopa equivalent daily dose was calculated using the formula reported by Tomlinson et al. (24).

The Edinburgh Handedness Inventory and neuropsychological tests were also administered on the day of testing to all participants including the Montreal Cognitive Assessment test (25) and Mini Mental State Examination (26) to rule out dementia, Spielberger State and Trait Anxiety Inventory (27), and Beck Depression Inventory-II (28). The Fatigue Severity Scale (29) was administered only to patients.

### Dynamic Isometric Task

The SE and its response to dopaminergic treatment and VF were tested in a dynamic isometric task using a hand clench dynamometer (Biopac Systems, Inc.) (Figure S1 in Supplementary Material). This is a rigid device that weighs 323 g, is 17.78 cm × 5.59 cm × 2.54 cm in size, and has an isometric range of 0–90 kg. It measures the applied force in voltage which is then converted to kilograms (nominal output: 782 μV/kg).

Participants sat comfortably in a chair in front of a computer holding the dynamometer in their hand with their forearm supported. The grip force of both hands was measured separately at the beginning of each test session. Participants were instructed to give their full grip force for 3 s, and the maximum voluntary contraction (MVC) was calculated as the average of 10 peak values. Fifty percent of the MVC was computed (MVC50) and used as the target force. Participants squeezed the dynamometer with each hand, repetitively, at MVC50 paced by a metronome cue at 1.25 Hz. Movement velocity covaries with the exerted force. By using external pacing, we aimed to keep the movement rate steady and examined the decrement in force across repetitions. The 1.25 Hz frequency was chosen based on previous studies (30) and our pilot data that showed that patients were able to match it.

There were two additional conditions: (1) all participants performed the task without VF (VF−) by solely relying on their perception of effort required to reach the MVC50, and then with VF (VF+) by monitoring their performance on the computer display. (2) Patients were tested twice, first in off- then in on-dopaminergic state at least 1 h apart.

The VF item had the shape of a speedometer. The midpoint represented the MVC50 target. In the VF+ condition, the needle of the speedometer moved up toward the target in real time with participant’s each squeeze. In the VF− condition, needle movement toward the target was simulated by the computer. This was done to match the visual input in both conditions, and participants were aware that the computer display did not reflect their performance in the VF− condition.

Initially, all participants received training at MVC50 with VF to form an internal representation of the required force for MVC50. After training, the VF− session started. The VF+ session followed immediately after the VF− with the same hand. Each session lasted 90 s. In the end of each session, the MVC was measured again to obtain a measure of fatigue.

### Modeling the SE

The first squeeze was discarded as it was highly variable and seemed to reflect participants’ attempt to calibrate their force. The rest of the dataset was smoothed using a sliding window of five time points. Previous studies and our pilot data demonstrated that the decrement requires ~15 s to occur (8, 30, 31). For consistency, we chose the first 20 squeezes (after the first was discarded) as the segment to examine for SE (short segment). This segment corresponded to ~15 s in all subjects (Table S1 in Supplementary Material). The force applied in each squeeze was normalized to the initial MVC. The peak of each squeeze was extracted using the peakfinder.m code in Matlab 2013b. Then, a linear slope was fitted to these peaks. The whole 90 s segment corresponding to 100 squeezes was analyzed separately to evaluate overall fatigue.

We also computed the area under the curve (AUC) of each peak using the trapezoidal numerical integration function in Matlab with unit spacing from the previous minimum to the current maximum peak. The AUC is equivalent to the integrated force over time, which corresponds to the motor impulse. We defined the motor energy as the capacity to create the motor impulse and used the sum of AUC across 20 squeezes as a measure of total motor energy expended during the short segment. The sum of AUC across the whole segment was analyzed separately. The change in AUC across each squeeze was also computed (Supplementary Material).

### Statistical Analyses

The primary outcome measures were the slope values fitted to the peaks and the sum of AUC.

#### Slope and Sum of AUC

A critical observation was that the initial force, which was supposed to be MVC50, varied considerably between groups, therefore, had to be included in the main analyses as a covariate. To compare the primary outcome measures slope and sum of AUC between the groups, we performed ANCOVAs with dependent variables slope and sum of AUC, fixed factors (1) group (HV vs. PD-off, HV vs. PD-on, and PD-off vs. PD-on), (2) hand (left/right), and (3) feedback (VF−/VF+); and initial force as the covariate. These analyses were employed for both short and whole segments separately.

#### Initial Force

Additionally, we used a repeated measure ANOVA to compare the initial force (i.e., the second squeeze) values between HV vs. PD-off, HV vs. PD-on, and PD-off vs. PD-on using feedback (VF+/VF−) and hand (left/right) as within-subject, and group as between-subject factors.

#### Maximum Voluntary Contraction

The first MVC (MVC1) and final MVC (MVC2) values were also compared with the same repeated measures ANOVA approach used for the initial force analysis. This analysis was performed to assess overall fatigue at the end of 90 s.

#### Clinical

The Montreal Cognitive Assessment test, Mini Mental State Examination, Spielberger State and Trait Anxiety Inventory, and Beck Depression Inventory-II scores were compared using two-sample *t*-tests between the PD and HV groups. The clinical measures, including the UPDRS total, UPDRS-III motor exam, Spielberger State and Trait Anxiety Inventory, Beck Depression Inventory-II, and Fatigue Severity Scale, were also used as regressors in a multiple regression analysis to assess their correlations with the dependent variables peak slopes and sum of AUC of the short segment during VF− and VF+ conditions in PD-off and PD-on.

All statistical analyses were performed using SPSS version 22.

## Results

### Clinical

Demographic and clinical data are summarized in Table 1. The average levodopa equivalent daily dose for the PD group was 932 ± 514 mg. The average Montreal Cognitive Assessment test score was 28.3 ± 2.3 in the HV and 27.9 ± 1.9 in the PD group. The average Mini Mental State Examination score was 29.5 ± 1.0 in the HV and 29.5 ± 0.9 in the PD group. The average Spielberger State and Trait Anxiety raw scores were 22.2 ± 2.8 and 26.8 ± 7.3 in the HV group, respectively, and 29.3 ± 9.2 and 34.0 ± 10.2 in the PD group, respectively. These scores were within the score range of a normative sample between the ages 50 and 69 years (27). The average Beck Depression Inventory-II score was 1.9 ± 3.2 in the HV and 7.0 ± 3.1 in the PD group (0–13 indicates minimal depression) (28). The average Fatigue Severity Scale score for the PD group was 39.8 ± 11.4, which was above the cut-off 36 and indicated significant fatigue (29).

**Table 1:** **Demographic and clinical data**.

	Age	Sex	S/T	BDI	FSS	LEDD (mg)	H&Y off/on	UPDRS-t off/on	UPDRS-III off/on
HV1	62	M	27/–	0					
HV2	54	F	24/22	1					
HV3	52	F	20/23	9					
HV4	56	F	22/36	0					
HV5	68	F	22/37	2					
HV6	75	F	21/22	2					
HV7	57	F	20/21	0					
HV8	65	M	23/40	8					
HV9	61	M	28/28	1					
HV10	69	M	20/24	0					
HV11	67	M	20/22	0					
HV12	67	M	20/20	0					
PD1	66	M	21/–	7	33	1650	2/2	60/45	35/26
PD2	54	F	23/27	3	48	667	2/2	57/33	30/18
PD3	56	F	23/25	2	33	600	2.5/2.5	53/21	32/13
PD4	55	F	45/47	13	42	1093	3/3	52/43	28/19
PD5	68	M	25/29	8	25	2125	2.5/2	39/26	27/16
PD6	75	F	30/32	3	50	1083	2/2	42/32	28/21
PD7	58	F	33/38	9	50	975	2.5/2.5	51/38	33/26
PD8	66	M	30/26	6	38	670	2/2	50/38	31/24
PD9	63	M	20/31	9	45	220	2/2	41/37	26/22
PD10	70	M	49/37	8	58	700	2.5/2	65/56	39/32
PD11	63	M	24/25	9	37	700	2/2	64/59	42/38
PD12	62	M	29/57	7	18	700	2/2	52/41	33/26

*HV, healthy volunteers; PD, Parkinson’s disease patients; S/T: Spielberger Anxiety Inventory State/Trait; BDI, Beck Depression Inventory-II; FSS, Fatigue Severity Scale; LEDD, levodopa equivalent daily dose; H&Y, Hoehn and Yahr; UPDRS, Unified Parkinson’s Disease Rating Scale; t, total score; III, motor examination score; off/on, off/on dopaminergic medication; M, male; F, female*.

The comparison of these scores (*p* value adjusted for five comparisons: 0.05/5 = 0.01) revealed a significant difference in the BDI-II scores between PD and HV groups (*p* = 0.0007). There was a trend for significance in the state anxiety scores (*p* = 0.018).

The PD group had an average H&Y score of 2.25 ± 0.3 in the “off” and 2.17 ± 0.3 in the “on” state. The average total UPDRS score was 52.1 ± 8.5 in the “off” and 39.1 ± 10.9 in the “on” state. The average UPDRS-III motor exam score was 32.0 ± 4.8 in the “off” and 23.4 ± 6.9 in the “on” state.

### Behavioral

**Figure 1:**
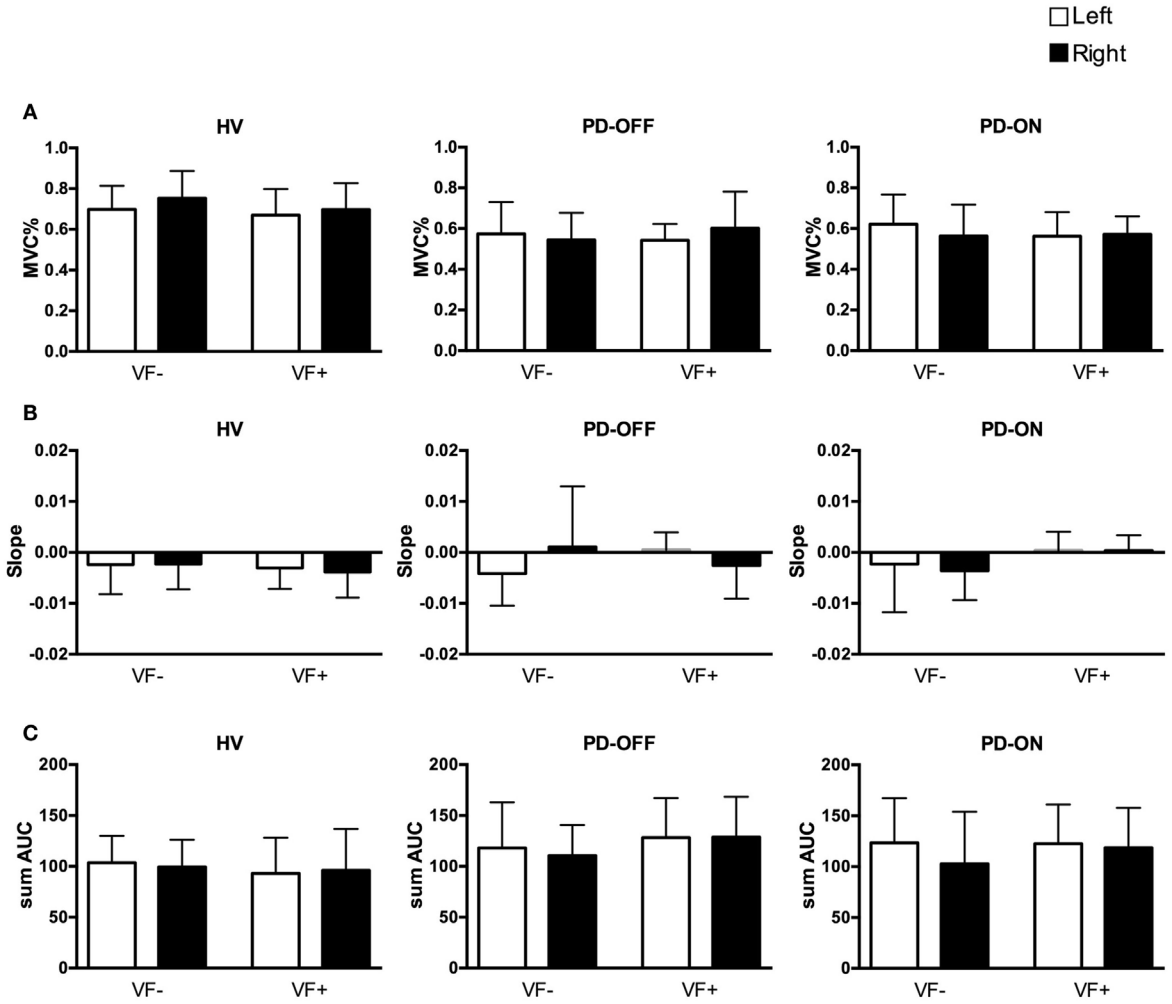
**Behavioral data of the short segment**. White columns: left hand, black columns: right hand. Error bars: SD. HV, healthy volunteers; PD, Parkinson’s disease patients; off/on, off/on dopaminergic medication; VF−/+, visual feedback, without and with; MVC, maximum voluntary contraction; sum AUC, sum of area under the curve. **(A)** Mean ± SD of the initial force as a percentage of the MVC. **(B)** Mean ± SD of slopes of the short segment. **(C)** Mean ± SD of the sum of AUC.

The mean and SD values of the initial forces, MVCs, and slope and sum of AUC values for the short segment are listed in Table 2 (see also Figure 1).

**Table 2:** **Behavioral data**.

	HV		PD-off		PD-on	
	L	R	L	R	L	R
**Initial force (% MVC)**						
VF−	0.69 ± 0.11	0.75 ± 0.13	0.57 ± 0.15	0.54 ± 0.13	0.62 ± 0.14	0.56 ± 0.15
VF+	0.67 ± 0.13	0.69 ± 0.13	0.54 ± 0.08	0.60 ± 0.18	0.56 ± 0.12	0.57 ± 0.09
**Slope**						
VF−	−0.002 ± 0.006	−0.002 ± 0.005	−0.004 ± 0.006	0.001 ± 0.012	−0.002 ± 0.009	−0.003 ± 0.006
VF+	−0.003 ± 0.004	−0.004 ± 0.005	0.000 ± 0.003	−0.002 ± 0.006	0.000 ± 0.003	0.000 ± 0.003
**Sum of AUC**						
VF−	103.5 ± 26.6	99.5 ± 26.8	118.1 ± 44.8	110.6 ± 30.0	123.4 ± 43.9	102.8 ± 51.0
VF+	93.1 ± 35.1	96.0 ± 40.7	128.2 ± 38.8	128.7 ± 39.6	122.7 ± 38.4	118.6 ± 39.1
**MVC1 (kg)**						
VF−	15.31 ± 6.67	16.02 ± 6.75	17.84 ± 8.16	18.74 ± 6.49	17.08 ± 6.23	18.00 ± 9.53
VF+	15.67 ± 6.62	15.62 ± 7.38	18.94 ± 6.79	18.41 ± 8.08	16.55 ± 6.37	18.56 ± 9.09
**MVC2 (kg)**						
VF−	15.95 ± 7.05	15.96 ± 7.16	16.16 ± 5.73	17.06 ± 6.82	17.46 ± 8.17	17.11 ± 8.92
VF+	14.78 ± 6.46	16.53 ± 7.25	15.88 ± 4.96	16.46 ± 6.02	15.84 ± 7.59	18.53 ± 9.23

*The mean and SD values of the initial force as percentage of the MVC, initial and final MVC, and slope and sum of AUC values for the short segment are shown*.

*HV, healthy volunteers; PD, Parkinson’s disease patients; off/on, off/on dopaminergic medication; L, left hand; R, right hand; AUC, area under the curve; MVC, maximum voluntary contraction; 1, initial; 2, final; VF−/+, visual feedback, without and with*.

*The slope and sum of AUC values belong to the short segment*.

#### Initial Force

Only a single significant main effect of group was observed in both HV vs. PD-off and HV vs. PD-on comparisons, namely, the HV group showed significantly higher initial force [*F*(1,22) = 12.974, *p* = 0.002 and *F*(1,22) = 10.001, *p* = 0.005, respectively]. There was no significant main effect of hand or feedback, and no interaction. The initial force did not differ significantly in the PD group between “off” and “on” states.

#### Short Segment

##### Peak Slopes

The ANCOVA in the HV vs. PD-off comparison revealed only a significant main effect of the initial force [*F*(8,87) = 38.947, *p* = 0.000]. In the HV vs. PD-on comparison, again a significant main effect of the initial force [*F*(8,87) = 49.709, *p* = 0.000], as well as a significant group × feedback interaction were observed [*F*(8,87) = 7.334, *p* = 0.008]. Planned pairwise comparisons revealed that the VF− condition was a significant factor [*F*(1,87) = 8.864, *p* = 0.004] and PD-on had significantly more negative slopes in the VF− condition compared to HVs [*F*(1,87) = 4.391, *p* = 0.04].

In the PD-off vs. PD-on comparison, the initial force demonstrated a significant main effect [*F*(8,87) = 56.43, *p* = 0.000], and there was a significant three-way interaction between medication status × feedback × hand [*F*(8,87) = 4.026, *p* = 0.048]. However, planned pairwise comparisons did not show significant differences.

##### Sum of AUCs

The ANCOVA in the HV vs. PD-off and HV vs. PD-on comparisons showed a significant main effect of the initial force [*F*(8,87) = 6.525, *p* = 0.012 and *F*(8,87) = 10.999, *p* = 0.001, respectively] and a significant main effect of the group [*F*(8,87) = 16.872, *p* = 0.000 and *F*(8,87) = 14.127, *p* = 0.000, respectively]. The PD group had a significantly higher sum of AUC in “off” and “on” states compared to the HV group. There was no significant main effect of hand or feedback, or any interaction.

In the PD-off vs. PD-on comparison, only the initial force showed a significant main effect [*F*(8,87) = 51.453, *p* = 0.000]. There was no significant main effect of medication status, hand or feedback, or any interaction.

#### Whole Segment

##### Peak Slopes

Both in the HV vs. PD-off and HV vs. PD-on comparisons, there was a significant main effect of the initial force [*F*(8,87) = 4.138, *p* = 0.045 and *F*(8,87) = 6.129, *p* = 0.015, respectively] and feedback [*F*(8,87) = 22.589, *p* = 0.000 and *F*(8,87) = 24.642, *p* = 0.000, respectively]. The slopes were less negative in the VF+ condition.

In the PD-off vs. PD-on comparison, there was only a significant main effect of feedback [*F*(8,87) = 34.145, *p* = 0.000] demonstrating less negative slope in the VF+ condition.

##### Sum of AUCs

In the HV vs. PD-off comparison, the initial force [*F*(8,87) = 9.297, *p* = 0.003], group [*F*(8,87) = 29.271, *p* = 0.000] and feedback [*F*(8,87) = 6.115, *p* = 0.015] showed significant main effects. There was no significant interaction between any factors. The sum of AUC was higher in the VF+ condition. PD-off showed higher sum of AUC in all conditions.

In the HV vs. PD-on comparison, there was a significant main effect of the initial force [*F*(8,87) = 6.503, *p* = 0.013], group [*F*(8,87) = 14.605, *p* = 0.000], and feedback [*F*(8,87) = 4.161, *p* = 0.044]. There was no significant interaction between any factors. The sum of AUC was higher in the VF+ condition. PD-on showed higher sum of AUC in all conditions.

In the PD-off vs. PD-on comparison, there was a significant main effect of the initial force [*F*(8,87) = 50.181, *p* = 0.000], feedback [*F*(8,87) = 10.723 *p* = 0.002], and hand [*F*(8,87) = 5.348, *p* = 0.023]. There was no significant interaction between any factors. The sum of AUC was higher in the VF+ condition, and the left hand demonstrated higher sum of AUC across all conditions.

#### MVC1 and MVC2

One patient could complete only 75 squeezes with left hand during “on” in the VF− condition and the matched HV did not have the MVC2 of the right hand in the VF− condition due to recording failure. Both subjects were excluded from the MVC analysis.

The MVC1 values did not differ significantly between the groups in the HV vs. PD-off, HV vs. PD-on, and PD-off vs. PD-on comparisons. There was also no significant main effect of feedback or hand, or any interactions. The MVC2 values also did not differ significantly between the groups in the HV vs. PD-off comparison. There was no significant main effect of feedback or hand, or any interactions. In the HV vs. PD-on comparison, there was a significant hand × feedback interaction [*F*(1,20) = 8.404, *p* = 0.009]. Pairwise comparisons showed that the left hand [*F*(1,20) = 8.058, *p* = 0.01] and the VF+ condition [*F*(1,20) = 10.467, *p* = 0.004] were significant factors demonstrating that the left hand MVC2 value was significantly smaller in the VF+ condition.

In the PD-off vs. PD-on comparison of the MVC2 values, there was a significant three-way interaction between hand × feedback × medication status [*F*(1,20) = 4.453, *p* = 0.048]. Planned pairwise comparisons showed significant medication status (PD-on) × feedback (VF+) [*F*(1,20) = 7.695, *p* = 0.012] and medication status (PD-on) × hand (left hand) [*F*(1,20) = 5.152, *p* = 0.034] interactions. Taken together, these interactions demonstrated significantly lower MVC2 in the left hand of PD-on during the VF+ condition.

#### Correlations with the Clinical Measures

The multiple regression analysis with the clinical measures was not significant for the peak slopes or the sum of AUC of the short segment during VF− or VF+ conditions in PD-off or PD-on.

## Discussion

Our main findings can be summarized as follows: (1) the groups were well matched in their MVC. (2) The magnitude of the initial squeeze played a significant role in the degree of the decrement. (3) HV group exhibited significantly higher initial force compared to PD-off and PD-on in all conditions. (4) PDs in the “on” state showed significantly higher SE compared to HVs in the VF− condition. (5) PDs demonstrated a higher sum of AUC for the short segment compared to HVs, and this was independent of feedback and medication status. (6) Both groups benefited comparably from feedback in the whole segment showing less decrement in force and also showed higher sum of AUC with feedback. (7) PDs demonstrated a higher sum of AUC for the whole segment compared to HVs independent of feedback and medication status.

Next, we interpret these findings and discuss their potential neurophysiological underpinnings.

### Energetic Cost and Dopamine

Contrary to our hypothesis, we did not observe significantly higher SE in PD-off compared to the HVs in the VF− condition. Instead, PD-on showed significantly higher SE in the VF− condition compared to HVs in line with our second hypothesis stating dopaminergic treatment would not improve SE.

The initial force is an important determinant of SE and was significantly higher in HVs in all conditions. However, initial force alone does not explain the lack of significant SE in PD-off compared to the HVs because PDs in “on” and “off” states were comparable with regard to the initial force. We think that the large variance in slope values in PD-off, especially concerning the right hand in the VF− condition (Supplementary Material), is probably the main factor that might obscure a true difference in SE means between HV and PD-off.

Reports on the role of dopaminergic treatment in reversing the SE vary according to the experimental design and outcome measures of interest. Behavioral studies using static force paradigms (e.g., hand-grip) demonstrated decline in force over time in PD patients (7, 32, 33). This decline improved with levodopa in some studies (32, 33), but not in others (7). Isotonic force tasks (e.g., finger tapping) revealed a rate-dependent decline in the amplitude of the movement, which showed no or minimal improvement with levodopa (30, 31). Decreased speed was found to improve with levodopa (8, 31); however, fatigue, defined as decrement in speed or amplitude, did not improve (8).

In our experiment, we controlled for the speed component by externally pacing each squeeze with a metronome at a pace which patients were able to maintain in “off” and “on” states. However, the decrement in force was not reversed with dopaminergic treatment. We propose the following explanations for this observation: (1) relatively high energetic cost of each squeeze for patients and (2) failure of dopaminergic treatment to energize repetitive squeezes over time.

(1) We defined motor energy as the capacity to create the motor impulse needed to reach the peak of each squeeze. Our results demonstrated that the energetic cost (i.e., sum of AUC) of performance in the short segment was significantly higher in PDs compared to HVs regardless of the actual motor output, feedback condition, or medication status. In the cost–benefit models of motor control, the energetic cost was introduced as a variable and considered a major determinant of speed in discrete movements (18, 19). Higher sensitivity to movement energy cost (i.e., reduced motor vigor) was shown in PD patients in discrete reaching tasks (18, 19). PD patients were able to make fast and accurate movements in a reaching task, but required more attempts to reach the speed criterion. This was interpreted as a higher sensitivity to movement energy cost in PD (18). Our results are in line with this interpretation and suggest that the energetic cost of maintaining motor performance at a steady pace over time is higher in PD patients compared to HVs.
(2) Dopaminergic treatment improves the motor cortical drive to the muscle (11, 12). Dopamine is also known to increase motor response vigor (e.g., speed, force) and energize behavior (34, 35). For instance, dopamine depletion in the nucleus accumbens or anterior cingulate cortex in rats made the animals choose low-effort actions to obtain food, but without altering their food preference or intake (36). Similarly, PD patients chose low-effort actions to obtain rewards which improved with dopaminergic medication (37). Moreover, Niv et al. proposed a model to account for the motor response vigor in the context of reward/effort tradeoff in free-operant behavior (38). In this model, the average rate of reward was encoded by tonic dopamine levels. For instance, when the average rate of reward is high (high tonic dopamine level), then one will move faster. On the other hand, if the average rate of reward is low (dopamine depletion), then there is no urgency to move faster. According to this model, one could argue that optimal tonic dopamine levels can invigorate motor response by improving the cost/benefit ratio. However, as our results demonstrate, dopamine replacement does not seem sufficient to overcome the energetic cost of a continuous motor task and prevent SE. We cannot separate out the role of tonic vs. phasic dopamine levels in our study because pharmacological treatment most likely influences both types of signaling. We think that the continuous task probably demands a high rate of dopamine signaling which cannot be sustained by an overall increase in dopamine levels following replacement. This also suggests that other neurotransmitter systems (e.g., serotonin) might be involved (39).

### Energetic Cost and Visual Feedback

We cannot rule out the potential role of peripheral fatigue during the performance of the short segment in PDs. However, several observations pertaining to the whole segment point to additional mechanisms that might be involved.

Patients and HVs did not differ significantly in their performance across the whole segment. Furthermore, the initial and final MVC values were also not significantly different between the groups suggesting that the task demands were comparable for both groups. In addition, both groups were able to improve their performance with feedback throughout the whole segment even though the VF+ runs immediately followed the VF− runs of the same (i.e., already “tired”) hand. In other words, excessive peripheral fatigue does not explain the SE in PD-on in the VF− condition during the short segment of the task. We think that the SE may reflect the difficulty in sustaining motor performance when the required effort has to be motivated and generated internally.

Initiation and sequential performance require an internally driven mechanism to prepare the emotive, motor and sensory apparatus (“cues”) (16). The limbic and motor basal ganglia–cortical loops may serve the integration of these “cues.” Activation in specific components of the basal ganglia-motor cortical circuits as well as in the amygdala has been demonstrated in the performance recovery phase of a demanding motor task (40). Therefore, it is conceivable that the disrupted integration in the basal ganglia–cortical loops in PD may lead to defective cue production for the subsequent set of responses and result in SE. As discussed in the previous section, performance has an energetic cost which needs to be balanced by motivation to be sustained. Taken within this cost–benefit context, the defective cue production might be the neural mechanism underlying the inability to overcome the cumulative energetic cost of a repetitive task (41).

The role of VF becomes particularly important at this point. In fact, VF+ reversed the SE in PD-on by providing an external reference. This result is consistent with previous reports of sustained improvement in stride length in PD patients in response to VF (4, 42, 43). One possible mechanism for this improvement is that VF directly facilitates the implementation of motor commands by the motor cortices *via* the parietal cortex (44). An alternative, but not mutually exclusive route, for VF might be the cerebellum. The cerebellum also integrates visual–motor information and plays a major role in the predictive timing and coordination of isometric grip forces (45). Finally, VF can also be considered a motivational/attentional cue because, despite the increased motor energy expenditure, VF drives continued performance by presenting the goal explicitly and allowing online monitoring of performance. It should be noted that the effect of VF was also observed in the performance of all subjects throughout the whole segment. All subjects improved and maintained their performance with VF despite the higher energetic cost associated with the VF+ condition, which was even higher for PDs. This finding is consistent with the observations that individuals may still be capable of carrying on the desired task depending on motivational/attentional factors (46).

Finally, we did not find a correlation between our primary outcome measures (slope and sum of AUC) and clinical measures of fatigue, mood, or disease severity. One explanation would be that these measures might be too broad and not sensitive enough to explain the SE. Alternatively, SE is a unique phenomenon that is at least partially independent of fatigue as we have demonstrated, and it is observed at every stage of disease including mild-to-moderate severity. Furthermore, mood may not have been a significant contributor to the SE because the depression scores of the patients were in the minimal depression range and the anxiety scores were within the normal range suggesting that our PD cohort did not have a significant mood disorder.

In conclusion, we think that SE in PD is a motoric manifestation of a complex phenomenon that includes energetic, attentional, and motivational factors. Dopaminergic treatment fails to maintain motor vigor across subsequent responses implicating the involvement of other neurotransmitters. The cumulative motor cost may be related to failure to incorporate limbic/motivational cues into the motor plan. VF bypasses this bottleneck by providing the necessary cues externally.

### Limitations

Two points should be kept in mind in interpreting the results of this study: (1) the unequal variances in performance between groups and (2) the initial force as a significant factor. The large variance in PD-off, especially in the VF− condition, may have obscured a difference in means. The initial force is a significant independent determinant of performance and ideally, should be controlled for in the experiment.

Furthermore, patients were tested twice on the same day always in the same order, first “off” and then “on” medication. However, there was at least an hour between the two sessions, and in light of our results, general fatigue or learning as confounding factors during “on” testing seems unlikely. Future studies should address these issues more directly by testing the HVs twice.

## Author Contributions

All authors contributed to the design. ST and AP collected and analyzed the data. All authors contributed to the interpretation of the results. ST drafted the manuscript. All authors revised it critically and provided final approval of the version to be published.

## Conflict of Interest Statement

The authors declare that the research was conducted in the absence of any commercial or financial relationships that could be construed as a potential conflict of interest.

## References

[1] Hallett M Bradykinesia: why do Parkinson’s patients have it and what trouble does it cause? Mov Disord (2011) 26(9):1579–81. 10.1002/mds.23730 21547949

[2] Kang SY Wasaka T Shamim EA Auh S Ueki Y Dang N The sequence effect in de novo Parkinson’s disease. J Mov Disord (2011) 4(1):38–40. 10.14802/jmd.11006 24868390 PMC4027704

[3] Ling H Massey LA Lees AJ Brown P Day BL. Hypokinesia without decrement distinguishes progressive supranuclear palsy from Parkinson’s disease. Brain (2012) 135(Pt 4):1141–53. 10.1093/brain/aws038 22396397 PMC3326257

[4] Iansek R Huxham F McGinley J. The sequence effect and gait festination in Parkinson disease: contributors to freezing of gait? Mov Disord (2006) 21(9):1419–24. 10.1002/mds.20998 16773644

[5] Chee R Murphy A Danoudis M Georgiou-Karistianis N Iansek R. Gait freezing in Parkinson’s disease and the stride length sequence effect interaction. Brain (2009) 132(Pt 8):2151–60. 10.1093/brain/awp053 19433440

[6] Kang SY Wasaka T Shamim EA Auh S Ueki Y Lopez GJ Characteristics of the sequence effect in Parkinson’s disease. Mov Disord (2010) 25(13):2148–55. 10.1002/mds.23251 20669182 PMC4782591

[7] Solomon NP Robin DA. Perceptions of effort during handgrip and tongue elevation in Parkinson’s disease. Parkinsonism Relat Disord (2005) 11(6):353–61. 10.1016/j.parkreldis.2005.06.004 16105745 PMC3523673

[8] Espay AJ Giuffrida JP Chen R Payne M Mazzella F Dunn E Differential response of speed, amplitude, and rhythm to dopaminergic medications in Parkinson’s disease. Mov Disord (2011) 26(14):2504–8. 10.1002/mds.23893 21953789 PMC3318914

[9] Berardelli A Rothwell JC Thompson PD Hallett M. Pathophysiology of bradykinesia in Parkinson’s disease. Brain (2001) 124:2131–46. 10.1093/brain/124.11.2131 11673316

[10] Hallett M Parkinson revisited: pathophysiology of motor signs. Adv Neurol (2003) 91:19–28. 12442661

[11] Brown P. Cortical drives to human muscle: the Piper and related rhythms. Prog Neurobiol (2000) 60(1):97–108. 10.1016/S0301-0082(99)00029-5 10622378

[12] Salenius S Avikainen S Kaakkola S Hari R Brown P. Defective cortical drive to muscle in Parkinson’s disease and its improvement with levodopa. Brain (2002) 125:491–500. 10.1093/brain/awf042 11872607

[13] Demirci M Grill S McShane L Hallett M A mismatch between kinesthetic and visual perception in Parkinson’s disease. Ann Neurol (1997) 41(6):781–8. 10.1002/ana.410410614 9189039

[14] Maschke M Gomez CM Tuite PJ Konczak J. Dysfunction of the basal ganglia, but not the cerebellum, impairs kinaesthesia. Brain (2003) 126:2312–22. 10.1093/brain/awg230 12821507

[15] Konczak J Corcos DM Horak F Poizner H Shapiro M Tuite P Proprioception and motor control in Parkinson’s disease. J Mot Behav (2009) 41(6):543–52. 10.3200/35-09-002 19592360

[16] Chaudhuri A Behan PO Fatigue and basal ganglia. J Neurol Sci (2000) 179(S1–2):34–42. 10.1016/S0022-510X(00)00411-1 11054483

[17] Friedman JH Brown RG Comella C Garber CE Krupp LB Lou JS Fatigue in Parkinson’s disease: a review. Mov Disord (2007) 22(3):297–308. 10.1002/mds.21240 17133511

[18] Mazzoni P Hristova A Krakauer JW. Why don’t we move faster? Parkinson’s disease, movement vigor, and implicit motivation. J Neurosci (2007) 27(27):7105–16. 10.1523/JNEUROSCI.0264-07.2007 17611263 PMC6794577

[19] Baraduc P Thobois S Gan J Broussolle E Desmurget M A common optimization principle for motor execution in healthy subjects and parkinsonian patients. J Neurosci (2013) 33(2):665–77. 10.1523/JNEUROSCI.1482-12.2013 23303945 PMC6704928

[20] Salimpour Y Mari ZK Shadmehr R. Altering effort costs in Parkinson’s disease with noninvasive cortical stimulation. J Neurosci (2015) 35(35):12287–302. 10.1523/JNEUROSCI.1827-15.2015 26338339 PMC4556793

[21] Hughes AJ Daniel SE Kilford L Lees AJ. Accuracy of clinical diagnosis of idiopathic Parkinson’s disease: a clinico-pathological study of 100 cases. J Neurol Neurosurg Psychiatry (1992) 55(3):181–4. 10.1136/jnnp.55.3.181 1564476 PMC1014720

[22] Fahn S Elton R Unified Parkinson’s Disease Rating Scale. In: Fahn S Marsden CD Calne D Goldstein M, editors. Recent Developments in Parkinson’s Disease. Florham Park, NJ: MacMillan Health Care Information (1987). p. 153–63.

[23] Hoehn MM Yahr MD Parkinsonism: onset, progression, and mortality. 1967. Neurology (1998) 50(2):318–34. 10.1212/WNL.50.2.318 9484345

[24] Tomlinson CL Stowe R Patel S Rick C Gray R Clarke CE. Systematic review of levodopa dose equivalency reporting in Parkinson’s disease. Mov Disord (2010) 25(15):2649–53. 10.1002/mds.23429 21069833

[25] Nasreddine ZS Phillips NA Bédirian V Charbonneau S Whitehead V Collin I The Montreal Cognitive Assessment, MoCA: a brief screening tool for mild cognitive impairment. J Am Geriatr Soc (2005) 53(4):695–9. 10.1111/j.1532-5415.2005.53221.x 15817019

[26] Folstein MF Folstein SE McHugh PR “Mini-mental state”. A practical method for grading the cognitive state of patients for the clinician. J Psychiatr Res (1975) 12(3):189–98. 10.1016/0022-3956(75)90026-6 1202204

[27] Spielberger C Manual for the State-Trait Anxiety Inventory. Palo Alto, CA: Consulting Psychologists Press (1983).

[28] Beck AT Beck Depression Inventory-II. San Antonio, TX: The Psychological Corporation (1997).

[29] Krupp LB LaRocca NG Muir-Nash J Steinberg AD. The fatigue severity scale. Application to patients with multiple sclerosis and systemic lupus erythematosus. Arch Neurol (1989) 46(10):1121–3. 10.1001/archneur.1989.00520460115022 2803071

[30] Stegemöller EL Allen DP Simuni T MacKinnon CD. Rate-dependent impairments in repetitive finger movements in patients with Parkinson’s disease are not due to peripheral fatigue. Neurosci Lett (2010) 482(1):1–6. 10.1016/j.neulet.2010.06.054 20599591 PMC2924741

[31] Espay AJ Beaton DE Morgante F Gunraj CA Lang AE Chen R. Impairments of speed and amplitude of movement in Parkinson’s disease: a pilot study. Mov Disord (2009) 24(7):1001–8. 10.1002/mds.22480 19230031

[32] Ziv I Avraham M Michaelov Y Djaldetti R Dressler R Zoldan J Enhanced fatigue during motor performance in patients with Parkinson’s disease. Neurology (1998) 51(6):1583–6. 10.1212/WNL.51.6.1583 9855505

[33] Lou JS Kearns G Benice T Oken B Sexton G Nutt J. Levodopa improves physical fatigue in Parkinson’s disease: a double-blind, placebo-controlled, crossover study. Mov Disord (2003) 18(10):1108–14. 10.1002/mds.10505 14534913

[34] Beierholm U Guitart-Masip M Economides M Chowdhury R Düzel E Dolan R Dopamine modulates reward-related vigor. Neuropsychopharmacology (2013) 38(8):1495–503. 10.1038/npp.2013.48 23419875 PMC3682144

[35] Salamone JD Pardo M Yohn SE López-Cruz L SanMiguel N Correa M Mesolimbic dopamine and the regulation of motivated behavior. Curr Top Behav Neurosci (2016) 27:231–57. 10.1007/7854_2015_383 26323245

[36] Salamone JD Correa M. The mysterious motivational functions of mesolimbic dopamine. Neuron (2012) 76(3):470–85. 10.1016/j.neuron.2012.10.021 23141060 PMC4450094

[37] Chong TT Bonnelle V Manohar S Veromann KR Muhammed K Tofaris GK Dopamine enhances willingness to exert effort for reward in Parkinson’s disease. Cortex (2015) 69:40–6. 10.1016/j.cortex.2015.04.003 25967086 PMC4533227

[38] Niv Y Daw ND Joel D Dayan P. Tonic dopamine: opportunity costs and the control of response vigor. Psychopharmacology (Berl) (2007) 191(3):507–20. 10.1007/s00213-006-0502-4 17031711

[39] Pavese N Metta V Bose SK Chaudhuri KR Brooks DJ. Fatigue in Parkinson’s disease is linked to striatal and limbic serotonergic dysfunction. Brain (2010) 133(11):3434–43. 10.1093/brain/awq268 20884645

[40] Bonzano L Tacchino A Saitta L Roccatagliata L Avanzino L Mancardi GL Basal ganglia are active during motor performance recovery after a demanding motor task. Neuroimage (2013) 65:257–66. 10.1016/j.neuroimage.2012.10.012 23063450

[41] Boksem MA Tops M. Mental fatigue: costs and benefits. Brain Res Rev (2008) 59(1):125–39. 10.1016/j.brainresrev.2008.07.001 18652844

[42] Morris ME Iansek R Matyas TA Summers JJ. Stride length regulation in Parkinson’s disease. Normalization strategies and underlying mechanisms. Brain (1996) 119:551–68. 10.1093/brain/119.2.551 8800948

[43] Morris ME Iansek R Galna B. Gait festination and freezing in Parkinson’s disease: pathogenesis and rehabilitation. Mov Disord (2008) 23:S451–60. 10.1002/mds.21974 18668618

[44] Vaillancourt DE Thulborn KR Corcos DM. Neural basis for the processes that underlie visually guided and internally guided force control in humans. J Neurophysiol (2003) 90(5):3330–40. 10.1152/jn.00394.2003 12840082

[45] Manto M Haines D. Cerebellar research: two centuries of discoveries. Cerebellum (2012) 11(2):446–8. 10.1007/s12311-011-0336-4 22113501

[46] Lou JS. Physical and mental fatigue in Parkinson’s disease: epidemiology, pathophysiology and treatment. Drugs Aging (2009) 26(3):195–208. 10.2165/00002512-200926030-00002 19358616

