# Supplementary material for: Sequence Effect in Parkinson’s Disease Is Related to Motor Energetic Cost
Source: Front Neurol. 2016 May 24;7:83. doi: 10.3389/fneur.2016.00083 (PMC4877367; doi:10.3389/fneur.2016.00083)
Supplement: Supplementary file 1 [file Data_Sheet_1.DOCX]

**1. Variance analysis**

Variance of slope values of the short segment for each hand, feedback condition, and group was calculated and F-ratios were computed.

The critical value for df = 11 (alpha = 0.025, two-tailed) is 3.53. Therefore, the PD-off/HV F-ratio of 5.68 for the right hand in the VF- condition indicated a significantly larger variance.

The PD-off/PD-on F-ratio of 4.23 for the right hand in the VF- condition and that of 4.79 for the right hand in the VF+ condition were also significant.

The F-ratios for other comparisons were not significant.

**2. Analysis of change in AUC**

We fitted a linear slope to the AUC of each peak for the short and whole segments. We performed ANCOVAs with dependent variables slope of the AUC, fixed factors 1) group (HV vs PD-off, HV vs PD-on, and PD-off vs PD-on), 2) hand (left/right), and 3) feedback (VF-/VF+); and initial force as the covariate. These analyses were employed for both short and whole segments separately.

**2.1. Short segment**

The ANCOVA in the HV vs PD-off comparison revealed a significant main effect of the initial force (F(8,87) = 9.148, p = 0.003), as well as a significant group x feedback interaction (F(8,87) = 4.07, p = 0.04). Planned pairwise comparisons showed that the VF+ condition was a significant factor (F(1,87) = 5.180, p = 0.025) and HVs had significantly more positive slopes in the VF+ condition compared to PD-off (F(1,87) = 14.875, p = 0.000).

In the HV vs PD-on comparison, again a significant main effect of the initial force (F(8,87) = 10.607, p = 0.002) and a significant main effect of feedback (F(8,87) = 17.088, p = 0.000) were observed. Both groups showed significantly more positive slopes in response to VF. There were no interactions.

Similarly, in the PD-off vs PD-on comparison, a significant main effect of the initial force (F(8,87) = 22.336, p = 0.000) and a significant main effect of feedback (F(8,87) = 4.207, p = 0.043) were observed. Patients in “on” and “off” states showed significantly more positive slopes in response to VF. There were no interactions.

**2.2. Whole segment**

In the HV vs PD-off comparison, a significant main effect of feedback was observed (F(8.87) = 6.779, p = 0.011). Both groups showed significantly more positive slopes in response to VF. There were no interactions.

In the HV vs PD-on comparison, a significant group x hand interaction was observed (F(8,87) = 4.049, p = 0.047). However, planned pairwise comparisons showed neither group nor hand alone was a significant factor.

In the PD-off vs PD-on comparison, no significant main effect or interaction was observed.

**Table 1.** Timing of the first 20 squeezes

|  | Left VF- | Left VF+ | Right VF- | Right VF+ |
| --- | --- | --- | --- | --- |
| HV | 15.2 ± 0.1 | 15.1 ± 0.3 | 15.0 ± 0.2 | 15.2 ± 0.2 |
| PD-off | 15.1 ± 0.2 | 15.1 ± 0.4 | 15.2 ± 0.2 | 15.2 ± 0.5 |
| PD-on | 15.2 ± 0.3 | 15.2 ± 0.2 | 15.2 ± 0.2 | 15.2 ± 0.7 |

Mean ± SD in seconds. HV: Healthy volunteer, PD-off/on: Parkinson’s patients in off-/on-medication states. ANOVA tests using group as between-subject and hand (left/right) and feedback (VF-/VF+) as within-subject factors did not reveal any significant differences. There was no significant main effect of group, hand, or feedback, and no significant interactions.
