# Supplementary figures and images for: Sequence Effect in Parkinson’s Disease Is Related to Motor Energetic Cost
Source: Front Neurol. 2016 May 24;7:83. doi: 10.3389/fneur.2016.00083 (PMC4877367; doi:10.3389/fneur.2016.00083)

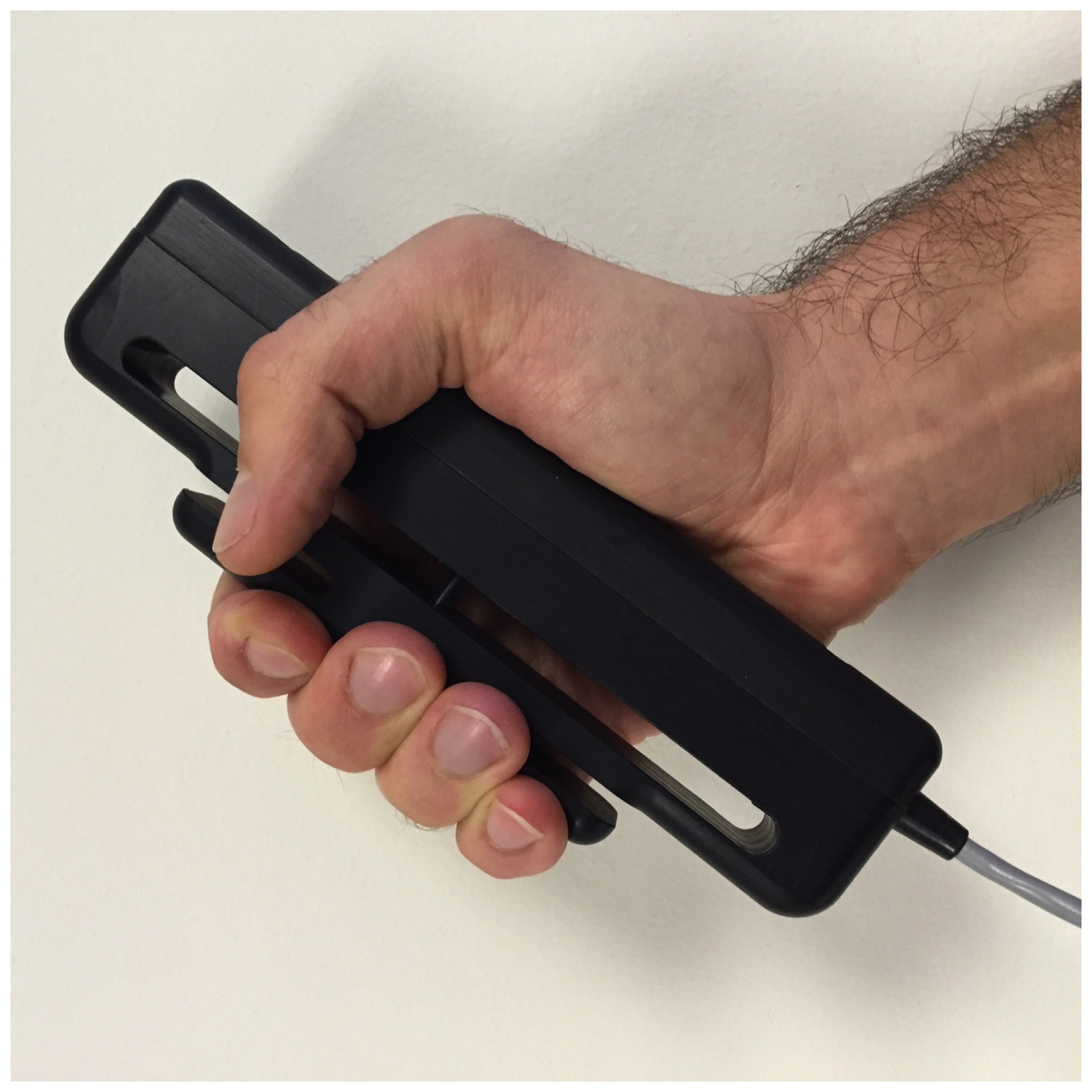

Supplement: Supplementary file 2 [file Image_1.JPEG]
